# PEST-CHEMGRIDS, global gridded maps of the top 20 crop-specific pesticide application rates from 2015 to 2025

**DOI:** 10.1038/s41597-019-0169-4

**Published:** 2019-09-12

**Authors:** Federico Maggi, Fiona H. M. Tang, Daniele la Cecilia, Alexander McBratney

**Affiliations:** 1 Environmental Engineering, School of Civil Engineering, The University of Sydney, 2006 Sydney, NSW Australia; 2 Sydney Institute of Agriculture, The University of Sydney, 2006 Sydney, NSW Australia

**Keywords:** Environmental sciences, Biogeochemistry

## Abstract

Available georeferenced environmental layers are facilitating new insights into global environmental assets and their vulnerability to anthropogenic inputs. Geographically gridded data of agricultural pesticides are crucial to assess human and ecosystem exposure to potential and recognised toxicants. However, pesticides inventories are often sparse over time and by region, mostly report aggregated classes of active ingredients, and are generally fragmented across local or government authorities, thus hampering an integrated global analysis of pesticide risk. Here, we introduce PEST-CHEMGRIDS, a comprehensive database of the 20 most used pesticide active ingredients on 6 dominant crops and 4 aggregated crop classes at 5 arc-min resolution (about 10 km at the equator) projected from 2015 to 2025. To estimate the global application rates of specific active ingredients we use spatial statistical methods to re-analyse the USGS/PNSP and FAOSTAT pesticide databases along with other public inventories including global gridded data of soil physical properties, hydroclimatic variables, agricultural quantities, and socio-economic indices. PEST-CHEMGRIDS can be used in global environmental modelling, assessment of agrichemical contamination, and risk analysis.

## Abstract (summary)

**Table:** 

Design Type(s)	modeling and simulation objective • data integration objective • statistical analysis and modeling objective
Measurement Type(s)	crop • pesticide
Technology Type(s)	statistical data analysis • computational modeling technique
Factor Type(s)	soil • hydroclimate • agricultural feature • Socioeconomic Indicator
Sample Characteristic(s)	United States of America • agriculture • Earth (Planet) • pasture • Europe

Machine-accessible metadata file describing the reported data (ISA-Tab format)

## Background & Summary

The amount and diversity of pesticides used in agriculture and horticulture have enormously increased since the Green Revolution to protect or increase yield, and enhance harvest and processing efficiency by the agro-food industry. The FAO reports 4.1 million tonnes of substances applied globally in 2015, that is, 35% greater than in 2000^1^. For the projected 9.77 billion people in 2050^2^ and the expected land conversion into arable production, global pesticide applications are likely to increase. Currently, the USGS and the European Commission list about 500 approved active ingredients, which may differ from country to country and can span across 18 pesticide classes. Regulatory authorities approve pesticides that do not pose toxicological health risk or are considered tolerable or non-persistent at the application rates recommended by manufacturers; however, there is evidence of pesticides residues in the environment^3,4^. Some pesticides can pose negative impacts on terrestrial and aquatic biodiversity even at sublethal doses such as triazines herbicides (e.g., atrazine) on the sexual development of amphibians^5^, the pre-emergence herbicide trifluralin on aquatic life^6^, or more recently, neonicotinoid insecticides (e.g., chlothianidin) on the immune system of bees^7^. Other organophosphate pesticides have been argued to potentially alter the biodiversity and shape ecosystem functions either when intensively used over large surface areas such as the non-selective herbicide glyphosate^8^ or when their toxicity persists for long periods such as the glyphosate degradation byproduct AMPA^9^, or other legacy pesticides such as DDT. Mixtures of common pesticides were shown to decrease the microbial species richness in laboratory samples by 15 to 30%^10^, while global scale analyses suggest an overall biodiversity decline due to loss in pollinators^11^ and land use-associated perturbations including the release of pollutants^12^.

There is therefore a pressing need, as recently highlighted by the Lancet Commission on Pollution and Health^13^, to specifically broaden our understanding of the global-scale use of pesticides and associated impacts on human health. Of the applied pesticides mass, the fraction not degraded chemically or biologically leads to residues that can be found practically everywhere including the atmosphere^14^, soil and water^4^, foods^15^, and even in remote regions far from intensive agriculture such as the Antarctic^16^. These residues can in principle become a hazard to humans; recent studies have shown that human intake of mixtures of pesticide residues (especially insecticides) at concentrations below the safety limit can have developmental and behavioural neurological effects^17,18^ and cause impairment of the endocrine system^19^, while populations exposed to some organophosphate pesticides (e.g., chlorpyrifos) suffered reduced thyroidal function^20^. The available databases of pesticide applications in some high-income countries have been particularly useful to monitor the environmental quality and find correlations with the epidemiology of emerging diseases in communities exposed to high levels of some active ingredients^21^. The ideal scenario would be to analyse human exposure to pesticides over scales spatially large enough to identify statistically significant correlations and achieve a global view on human health risks. However, this is currently limited by a lack of information of the geographic distribution of pesticide use.

Reality is that inventories of pesticide use compiled in most high- and middle-income countries are generally affected by missing data, such as time records, geographic locations, or active ingredients. Low-income countries rarely have a record of pesticides use. In addition, because databases are generally maintained by various independent authorities, they are fragmented and may be structured with incompatible formats and naming that hampers their fusion. The United Nations FAOSTAT pesticide database^1^ is the unique example that aims to harmonize and distribute worldwide data. However, yearly data are sorted by country and aggregated by pesticide class rather than by the individual active ingredient. In contrast, the database produced by the USGS Pesticide National Synthesis Project (USGS/PNSP) within the National Water Quality Assessment (NAWQA)^22^ includes the annually applied pesticide mass for more than 500 active ingredients, but it is specific to the USA. While both databases suffer from missing data, they together allow for a re-analysis and data reconstruction, and for validation of globally-gridded crop-specific pesticide annual application rates (mass per area per year); these data are currently not available in a coherent structure but are the ones with the greatest scope for multiple applications in environmental sciences and management, public health, modelling, and data-driven governance development.

The PEST-CHEMGRIDS database released here is a comprehensive global estimate of the 20 most used pesticide active ingredients applied to 6 dominant crops (corn, soyabean, wheat, cotton, rice, and alfalfa) and 4 aggregated crop classes (vegetables and fruit, orchards and grapes, pasture and hay, and others) at 5 arc-min resolution (about 10 km at equator). The estimates for 2015 are projected to 2025 using 25-year historical trends in the USA, and conditioned to country-specific governances on bans and approvals, regulations on genetically modified (GM) crops and GM-resistant ingredients, and pesticide class inventories of the FAOSTAT. The USGS/PNSP and FAOSTAT pesticide databases have been re-analysed and intersected with a number of other public inventories to estimate the pesticide application rates at global scales using statistical methods. The most important databases used for these estimates include global gridded data of soil physical properties, hydroclimatic and agricultural variables, and socio-economic metrics along with other corollary databases specified in Methods.

The objectives of creating PEST-CHEMGRIDS are: (i) to expand to a global extent the estimated annual application rates of major active ingredients used in dominant and aggregated crops from 2015 to 2025; (ii) to provide freely accessible validated inputs that can serve the modelling of environmental processes and risk assessments analyses from local to continental scales; and (iii) to raise the attention of the scientific and public communities on the pressing issue of contamination by agrochemicals. Released data are provided in standard globally-gridded formats and can ideally be coupled to any existing georeferenced layers.

## Methods

**Fig. 1:**
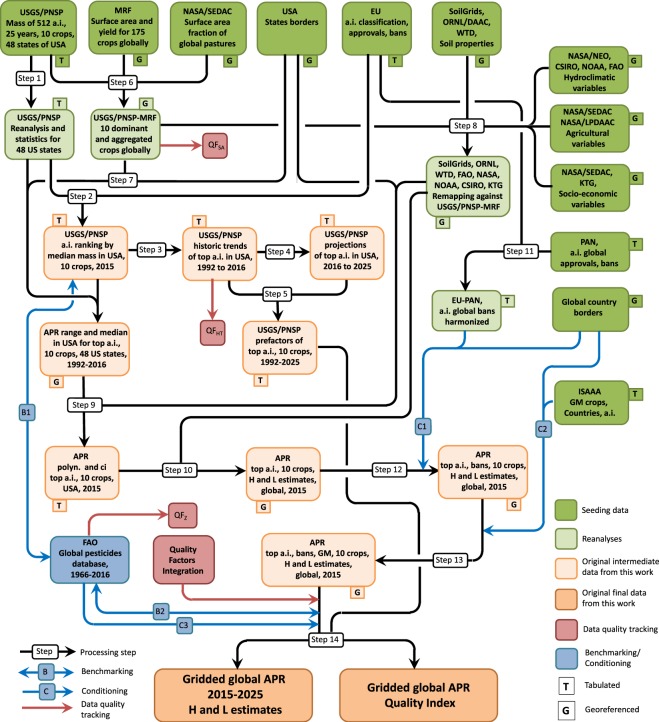
Flow chart. Processing steps implemented to elaborate source data sets and produce globally gridded yearly application rates of the top 20 crop-specific pesticides and their quality index maps.

Development of the PEST-CHEMGRIDS data release required the use of multiple publicly available data sources referenced in Table 1, and the development of several computational scripts to intersect and perform calculations on those data along various sequential and parallel steps. The overall workflow to generate the global maps of pesticides annual application rates and data quality is described in the following sections and is schematically represented in Fig. 1.

**Table 1:** Details and characteristics of source data sets.

Data source	Description	Details	Stored as	Ref.
FAOSTAT	Global pesticide use aggregated by country and pesticide class	Tabulated Expressed in [tonnes] from 1992 to 2016	.CVS	^1^
USGS/PSNP	Mass of 512 a.i. used from 1992 to 2016 is 48 USA states.	Tabulated Last updated in 2017.	.TXT	^22^
EU pesticide classification	A.i. tagged by class, approval and ban within the European Community	Tabulated Last updated on September 2016.	.XLS	^27^
MRF	Surface area and yield for 175 crops	Surface area expressed in [ha] and yield in [kg/ha] globally gridded at 5 arc-min resolution (10 km at the equator) estimated in year 2000	.TIF (georeferenced)	^28^
NASA/SEDAC	Land surface fraction used for pastures and hays.	Globally gridded at 5 arc-min resolution (about 10 km at the equator) in 2000	.TIF (georeferenced)	^29^
Administrative borders	USA states	Gridded at 0.0378 deg (about 4.5 km at the equator) in circa 2017	.SHP (georeferenced)	^30^
	All countries	Gridded at 0.082 deg (about 5 km at equator)	.SHP (georeferenced)	^48^
SoilGrids	Soil textural fraction (sand, silt, clay), porosity, and SOC in six layers from surface to 2 m depth.	Textural fractions expressed in percent, porosity expressed in percent, and SOC expressed in [g-C/kg-soil] globally gridded at 7.5 arc-sec resolution (250 m at the equator)	.TIF (georeferenced)	^31^
ORNL/DAAC	Thickness of soil, regolith and sedimentary layers	Expressed in [m] globally gridded at 30 arc-sec resolution (1 km at the equator)	.TIF (georeferenced)	^32^
WTD	Equilibrium water table depth	Expressed in [m] globally gridded at 15 arc-min resolution (30 km at the equator)	.NC v4 (georeferenced)	^33^
NOAA/NCEI	Daily precipitation	Expressed in [mm] at 15 arc-min resolution (30 km at theequator).	.NC v4 (georeferenced)	^34^
NOAA/NCEI	Daily atmospheric temperature	Expressed in [°C] at 15 arc-min resolution (30 km at the equator).	.NC v4 (georeferenced)	^36^
NOAA/NCEI	8-day net solar radiation	Expressed in [W/m^2^] at 15 arc-min resolution (30 km at the equator).	.TIF (georeferenced)	^37^
NASA/NEO	8-day net primary productivity	Expressed in [g-C/m^2^ day] globally gridded at 12 arc-min spatial resolution (about 25 km at the equator)	.TIF (georeferenced)	^38^
CSIRO	Monthly evapotranspiration	Expressed in [mm] at 0.5 degree resolution (about 55 km at the equator).	.NC v4 (georeferenced)	^39^
FAO Geonetwork	Thermal climatic regions	Classification of climates globally gridded at 1.25 arc-min resolution (2.5 km at the equator)	.TIF (georeferenced)	^40^
NASA/SEDAC	N fertilizer application rates	Expressed in [kg-N/ha year] globally gridded at 30 arc-min resolution (about 30 km at equator)	.TIF (georeferenced)	^41^
NASA/SEDAC	P fertilizer application rates	Expressed in [kg-P/ha year] globally gridded at 30 arc-min resolution (about 30 km at the equator)	.TIF (georeferenced)	^42^
NASA/LPDAAC	Crop water security describing irrigated and rain fed crops	Expressed by classification ranking and globally gridded at 5 arc-min resolution (10 km at the equator) in circa 2010	.TIF (georeferenced)	^44^
NASA/SEDAC	Population count and density	Expressed in [capita] and [capita/km^2^] globally gridded at 2.5 arc-min resolution (about 5 km at the equator) in circa 2015	.NC v4 (georeferenced)	^45^
KTG	Gross domestic product (GDP) and human development index (HDI)	GDP expressed in [int. USD] and HDI index globally gridded at 5 arc-min resolution (about 10 km at the equator) in circa 2015	.TIF (georeferenced)	^46^
PAN	List of banned pesticides	Tabulated Last update in May 2019	.XLS	^47^
ISAAA	Registry of approved GM crops and GM-specific pesticides by country	Tabulated Last updated in May 2019	.CVS	^60^

All major datasets are listed with key information on data type, units and format with which they are stored in public repositories.

### Acquisition of seeding databases and re-analysis (Step 1)

The globally-gridded pesticide application rates in PEST-CHEMGRIDS were estimated based on the USGS Pesticide National Synthesis Project (USGS/PNSP) within NAWQA^22^. The “high” and “low” annual application mass compiled for each state of the USA from 1992 to 2016 and for a total of 512 active ingredients relied on surveys used in conjunction with the USDA National Agricultural Statistics Service (USDA/NASS)^23^ for various years from 2007 to 2012, and interpolation and extrapolation methods originally described in^24^ when data were not available. The USGS/PNSP data are explicit for 6 dominant crops (i.e., corn, soyabean, wheat, cotton, rice, and alfalfa) and 4 aggregated crop classes (i.e., vegetables and fruit, orchards and grapes, pastures and hays, and others), which include the crops listed in Table 2 column 2. We did not reconstruct missing data in the original USGS/PNSP database except when either the “high” or the “low” estimate was available; in those cases, the yearly-averaged “high”-to-“low” or “low”-to-“high” mass ratio for a specific crop and active ingredient was used as a factor for the available datum to retrieve the missing datum. The year 2015 was used as the reference throughout for PEST-CHEMGRIDS; hence, a linear interpolation of data in 2015 was implemented when data in 2014 and 2016 were available to retrieve the crop-specific application mass of an active ingredient in 2015. Next, the maximum value of the “high” estimate and the average of the “low” estimate for the mass of each active ingredient in each year were calculated for the 48 available USA states of the USGS/PNSP to represent the range of applied mass in each of the 25 years assuming that the actual lower bound is always zero when no pesticides are applied (Fig. 1, step 1). The states of Alaska and Hawaii, and other minor USA territories were not included in the original USGS/PNSP database. Along with the range of applied mass, the median mass *M* was calculated and used in the next steps described below.

**Table 2:** List of dominant and aggregated crops classes and matching.

Crop class	USGS/PNSP	PEST-CHEMGRIDS
Dominant	Corn	Corn, *Corn FOR
Dominant	Soyabean	Soyabean
Dominant	Wheat	Wheat
Dominant	Cotton	Cotton
Dominant	Rice	Rice
Dominant	Alfalfa	Alfalfa
Aggregated Vegetable and fruit (VegFru)	Artichokes, Asparagus, Avocados, Beans Peas Vegetable, Beans (snap, bush, pole, string, Lima), Beets, Berries, Blueberries, **Broccoli, **Brussels sprouts, **Bulb crops, Cabbage, Caneberries, **Cantaloupes, Carrots, Cauliflower, **Celery, Chicory, **Cole crops, **Collards, Cranberries, Cucumbers, **Cucurbits, Currants, **Daikon, Dry beans peas, Eggplant, **Eggplant peppers, **Escarole and Endive, Garlic, Gingerroot, **Guavas, Herbs, **Horseradish, **Kale, Lettuce, Melons, Okra, Onions, Other non-citrus fruit, **Parsley, Peas (Green, Sweet), Peppers, Pineapples, Potatoes, Pumpkins, **Radishes, **Rhubarbs, Roots tubers, Spinach, **Squash, Strawberries, **Sweet corn, Sweet potatoes, Tomatoes, Turnips, Vegetables (leafy, other), Watermelons	Artichokes, Asparagus, Avocados, *Peas, Beans, *Beans (string, broad, green), *Beets FOR, Berries, Blueberries, Cabbage, *Caneberries (Raspberries, Gooseberries), Carrots, Cauliflower, Chicory, Cranberries, Cucumbers, Currants, Eggplant, Garlic, Gingerroot, *Herbs (Spices NES), *Dry beans peas (Legumes NES, Lentil, Chickpea, Pigeonpea, Pulse NES, cowpea), Lettuce, Melons, Okra, *Onions (green and others), *Other non-citrus fruit (Bananas, Plantain, fruits NES), Peas (Green, Sweet), Peppers, Pineapples, Potatoes, Pumpkins, *Root tubers (Cassava, Root NES, Yautia, Yam) Spinach, Strawberries, Sweet potatoes, Tomatoes, *Turnips (forage), *Vegetable (other), Watermelons
	**19/58 = 0.327 (unmatched fraction) 0.673 (matched fraction)	*12/58 = 0.21 (partial match fraction) 0.79 (matched fraction)
Aggregated Orchards and Grapes (OrcGra)	Almonds, Apples, Apricots, Cherries, Chestnuts, Citrus (other), Dates, Figs, Grapefruit, Grapes, **Grapevines, Hazelnuts, Kiwifruit, **Kumquats, Lemons, Limes, Mangoes, Nuts (trees and other), Olives, Oranges, Papayas, Peaches, Pears, **Pecans, Persimmons, Pistachios, Plums, **Pomelike fruit other, Prunes, Stone-like fruit other, **Tangelos and Tangerines, Walnuts	Almond, Apples, Apricot, Cherries, Chestnuts, *Citrus (other), Dates, Figs, Grapefruit, Grape, Hazelnuts, Kiwifruit, Lemons, Limes, Mangoes, *Nuts (Nutmeg, Brazil, Cashew, Groundnut, Nuts NES), Olives, Oranges, Papayas, Peaches, Pears, Persimmons, Pistachios, Plums, *Prunes (sour cherry), Stone-like fruits NES, Walnuts
	**5/33 = 0.151 (unmatched fraction) 0.849 (matched fraction)	*3/33 = 0.091 (partial match fraction) 0.909 (matched fraction)
Aggregated Pasture and hay (PasHay)	Cropland for pasture, RPLongtermAcres, Fallow/FallowSummer, Forage/Fodder, Hay other, Idle cropland other, Lots farmstead other, other Tame hay, Pastureland, Pasture Range, Pasture Rangeland other	Pasture, *Cabbage FOR, *Carrots FOR, *Forage NES, *Rye FOR, *Sorghum FOR, *Swede FOR, *Vegetable FOR, *Vetch
Aggregated Other crops (Other)	Barley, Field and grass seed crops all, Flax, **Flaxseed, Hops, **Jojoba harvested, Mustard (seed), Oats (for grain), Oats Rye, **Peanuts, Rye (for grain), Rapeseed (Canola), Safflower, Sesame, Sorghum, Sorghum Milo, Sugar (beets, Cane), Sunflowers, Taro, Tobacco, Triticale, **Wildrice, **Woodland, Other crops	Barley, *Field and grass seed crops all (Mixed Grass, Grass NES, Poppy, Hemp, Hempseed, Jute, Jute like fiber, Fibres NES, Kapok fiber, Fonio, Kapok seed, Linseed, Mixed Grain), Flax, hops, *Mustard, *Oats (Cereal NES, Millet, Lupin, Buckwheat), *Rye, *Rapeseed (Oilseed FOR, Oilseed NES), Safflower, Sesame, Sorghum, *Sugar (beets, Cane, Sugar NES), Sunflower, Taro, Tobacco, Triticale, *Other crops (Agave, Anise, Areca, Bambara, Canaryseed, Carob, Cashewapple, Castor, Chili, Cinnamon, Clove, Clover, Cocoa, Coconut, Coffee, Coir, Greencorn, Gums, Karite, Kolanut, Mate, Mushroom, Oil palm, Peppermint, Pimento, Popcorn, Pyrethrum, Quince, Quinoa, Ramie, Rubber, Sisal, Tea, Tropical NES, Tung, Vanilla)
	**5/24 = 0.208 (unmatched fraction) 0.792 (matched fraction)	*7/24 = 0.291 (partial match fraction) 0.709 (matched fraction)

The crop list in the USGS/PNSP database is reconstructed with the crops listed under column “PEST-CHEMGRIDS” and originally sourced in^28^. The matched, partial match, and unmatched fractions are calculated and tracked in the quality factor *QF*_*SA*_ described in “Technical Validation”. FOR and NES stand for “forage” and “not elsewhere specified”.

### Selection of active ingredients samples (Step 2)

**Fig. 2:**
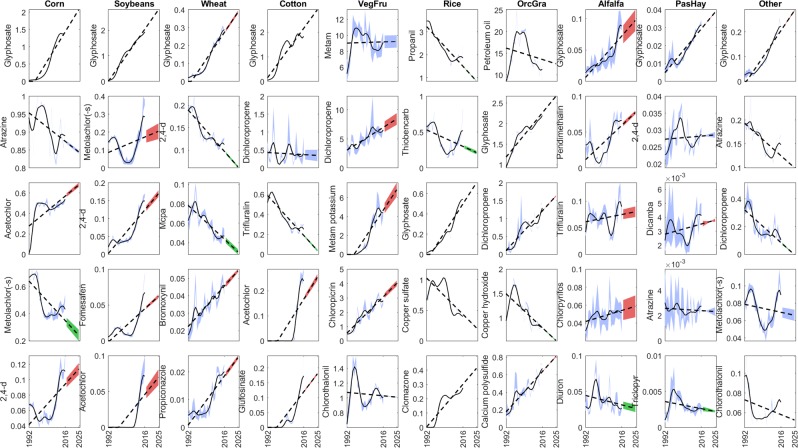
Top 5 of the 20 most used crop-specific active ingredients. The panels represents the “high” and “low” historical (within blue shaded areas from 1992 to 2016) and projected (dashed lines) application rates in kg/ha obtained for the 5 out 20 top active ingredients used on dominant and aggregated crops in the USA. Historical data are from the USGS/PNSP database while projections are obtained from step 4 in Fig. 1. Columns refer to dominant and aggregated crop classes. Shaded red, blue and green areas from 2016 to 2025 highlight active ingredients with increasing, steady and decreasing projection trends, respectively. Panels for the top 6 to 20 ingredients are available in^25,26^.

Using the reanalysis of the USGS/PNSP data, we ranked the active ingredients applied in the USA for each dominant and aggregated crop in decreasing order of mass (Fig. 1, step 2). Ranking was conducted by first integrating the applied median mass *M* over each state in the reference year 2015. Next, the top 20 most used crop-specific active ingredients were selected against satisfaction of the following conditions: (i) no more than 10 missing years over the 25-year records, and (ii) no more than 5 missing years in the last 10 years. In total, 95 different active ingredients were found, which cumulative mass represents 84.2% of the pesticide mass used in the USA in 2015 (see Fig. 2 for the top 5 out of 20 selected in each crop class, and^25,26^ for the full set of panels). This figure was validated against the FAOSTAT pesticide database (see “Technical Validation”) and the correction factor *F*_*M*_ = 0.842 was defined and later used in “Global conditioning against FAOSTAT pesticide records“ to account for the mass deficit after selection of active ingredients. Using the pesticide databases of the European Commission, which details 18 pesticide classes for more than 1300 substances as of 2019^27^, we determined that the selected active ingredients cumulatively include 7 pesticide classes distributed as 60 herbicides, 22 fungicides, 20 insecticides, 8 acaricides, 6 nematicides, 5 plant growth regulators and 1 repellent. Of these, 41 active ingredients belong to more than one pesticide class, and 1 was not classified. Seed treatments were included in our reanalysis as per the USGS/PNSP but we note that these have not been reported any longer from 2016 and 2017. The FAOSTAT database does not have data for seed treatments in the USA.

### Historical trends, projections, and mapping prefactors (Steps 3 to 5)

Raw historical trends of the median mass *M* of the selected active ingredients in the USA from 1992 to 2016 were first smoothed with cubic splines using the Matlab function *csaps*. The smoothed median masses *M*_*s*_ were next used to produce the first-order polynomial regressions (*M*_*r*_) as

1 $${M}_{r}(i,j,t)={a}_{i,j}\cdot t+{b}_{i,j},$$

where *t* represents time in year (Fig. 1, step 3). The dimensional polynomial parameters (*a*, *b*)_*i*,*j*_ specific to the selected active ingredient *i* on each dominant and aggregated crop *j* were recorded, totalling 200 couples. The projections in pesticide mass used in the USA were next calculated as *M*_*p*_(*i*, *j*, *t*_*p*_) = *M*_*r*_(*i*, *j*, *t* = *t*_*p*_) in years *t*_*p*_ ranging between 2015 and 2025 using Eq. (1) (Fig. 1, step 4) and are represented in Fig. 2 for selected active ingredients. The pesticide- and crop-specific projections *M*_*p*_ for years 2015 to 2025 were next normalized by the smoothed median mass *M*_*s*,2015_ in reference year 2015 to calculate the mapping prefactors (Fig. 1, step 5).

2 $$F(i,j,t)=\frac{{M}_{p}(i,j,{t}_{p})}{{M}_{s}(i,j,t=2015)}\,{\rm{for}}\,2015 < {t}_{p}\le 2025$$

Each pesticide- and crop-specific time-varying prefactor *F* calculated for the USA is a proportionality scalar used to condition the projections of pesticide application rates globally as described later. All polynomials coefficients along with goodness-of-fit metrics are distributed in^25,26^, while different methods to achieve time projections are discussed in “Validation of historical trends and projection prefactors”.

### Application rates in the USA (Steps 6 to 7)

The most important step at this stage was to convert the application mass of selected active ingredients into application rates (APR) expressed in mass per unit area (kg/ha per year). To this end, the global crop distribution database in^28^ was used (here called MRF as per the authors’ initials); this includes globally gridded harvested area (ha) and yield (kg/ha) for 175 crops estimated in year 2000. To match the MRF and UGSG/PNSP databases we rearranged the crops in the four aggregated crop classes (Fig. 1, step 6) using the aggregation list in Table 2, column 3. Crop aggregation using the MRF in^28^ matched the USGS/PNSP list by an average of 63.7% for Vegetable and Fruits (VegFru), 84.9% for Orchards and Grapes (OrcGra), and 79.2% for Other (Other) Crops. For the Pasture and Hay (PasHay) crop class, we used the global gridded pasture distribution of the NASA Socioeconomic Data and Applications Centre (NASA/SEDAC) inventory^29^, which reports the surface area fraction of grid cells used for general pastures in 2000 (Fig. 1, step 7). This layer was converted to area using the georeferencing projection details. For this crop class, we also included some crops from MRF that were explicitly tagged to forage, hence quantification of matching was not directly possible for the Pasture and Hay crop class used in the USGS/PNSP database. Corrections for the unmatched list of crop surface area at global scales were not implemented at this stage but as described in “Global conditioning against FAOSTAT pesticide records”. The fractions of matched crops were tracked and used as one of the data quality factors in the calculation of the quality index for this PEST-CHEMGRIDS release (see “Technical Validation”).

During assemblage of the dominant and aggregated crop classes we tested that the total area of all crops included in a grid cell did not exceed the grid cell area and we corrected where needed by introducing a maximum crop saturation of 0.95 in those cells. The rebuilt dominant and aggregated crops maps are therefore distributed with PEST-CHEMGRIDS in^25,26^ because they are slightly different from the disaggregated original maps in MRF in^28^ and because they may be needed for further processing of PEST-CHEMGRIDS by a third party. Table 3 reports the surface fraction of each dominant and aggregated crop after our manipulation.

**Table 3:** List of crop maps in the PEST-CHEMGRIDS release.

Crop class	Crop name	Fraction of total crop surface area
Dominant	Corn	0.04
	Soyabean	0.02
	Wheat	0.05
	Cotton	0.01
	Rice	0.03
	Alfalfa	0.01
Aggregated	Vegetables and fruits (VegFru)	0.04
	Orchards and grapes (OrcGra)	0.02
	Pastures and hays (PasHay)	0.68
	Other (Other)	0.1

Dominant and aggregated crops used in PEST-CHEMGRIDS defined by the crops in Table 2, column “PEST-CHEMGRIDS”, are corrected by the total surface area available in a grid cell. The original disaggregates crop layers are available in^28^. The PEST-CHEMGRIDS crop maps listed here are distributed in files equally stored in Portable Network Graphics (.PNG), Tagged Image File Format (.TIFF/.TIF), and NetCDF4 (.NC) formats in^25,26^.

The calculation of the historical APR relative to the USA states was ultimately accomplished by dividing the smoothed median application mass *M*_*s*_ by the area of each dominant and aggregated crops in each state for the selected active ingredients and each of the available 25 years of data records (Fig. 1, step 7). The georeferenced USA map from^30^ was used as a mask to identify the cumulative area of each crop class in individual USA states. Because we calculated the range and median application mass *M*_*s*_ earlier, the APR for the USA states was also expressed by a range and median.

### Global spatial inference of application rates (Steps 8 to 10)

The inference of pesticide application rates from the USA to global scales was conducted by means of a polynomial extrapolation from the 2015 APR in the USA states (determined in step 7) using 20 globally-gridded environmental quantities that included soil physical properties, hydroclimatic and agricultural variables, and socio-economic indices (Table 1). The procedures are implemented in Fig. 1, step 8 to 10, and are detailed below.

Soil physical properties were sourced from SoilGrids^31^, which consists of globally-gridded soil profiles through 6 layers from the surface to 2 m depth. For this work, we accessed the three soil textural fractions (sand, silt, and clay), the soil organic carbon content, and the soil porosity of the top layer. In addition to these, we also used the global soil thickness data of the Distributed Active Archive Centre for Biogeochemical Dynamics of the Oak Ridge National Laboratory (ORNL/DAAC)^32^ and the global equilibrium water table depth (WTD) by Fan *et al*.^33^.

Global hydroclimatic variables included daily precipitation from the CPC Global Unified Precipitation data provided by the NOAA/OAR/ESRL PSD, Boulder, Colorado, USA^34^, atmospheric temperature from the Global Historical Climatology Network - Daily (GHCN-Daily) dataset^35,36^, and the 8-day net solar radiation^37^ and the 8-day net primary productivity^38^ from the NASA Earth Observations (NASA/NEO), the monthly actual evapotranspiration available from the Commonwealth Scientific and Industrial Research Organization (CSIRO)^39^, and the thermal climate region maps of the FAO/GeoNetwork^40^.

Agricultural variables included the global annual application rate of nitrogen^41^ and phosphorous^42^ from NASA/SEDAC^43^, the yield of dominant and aggregated crops obtained from reanalysis of the USGS/PNSP and MRF data in step 6 at their original resolution (with the exception of the yield of pastures and hays sourced from NASA/SEDAC inventory)^29^, and the global crop water security (GFSAD) layer from NASA Land Processes Distributed Active Archive Center (NASA/LPDAAC)^44^.

Finally, socio-economic indices included the global population density map estimated in 2015 by^45^, and the gross domestic product (GDP) and human development index (HDI) maps in 2015 developed and distributed in^46^ (here called KTG from the authors’ initials).

**Fig. 3:**
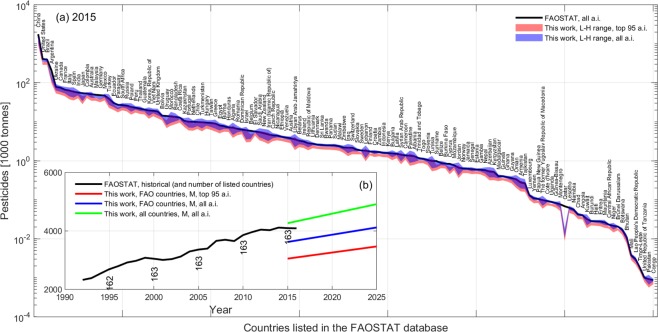
Benchmarking of conditioned estimates against the FAOSTAT pesticide data. (**a**) Aggregated pesticide use for all countries listed in the FAOSTAT database in 2015 and corresponding conditioned estimated for the top 95 and all active ingredients (a.i.), with the latter estimated using the correction factor *F*_*M*_ = 0.842. (**b**) Projected global pesticide mass for the countries listed in the FAOSTAT and all countries benchmarked against historical FAOSTAT records.

Data integrated in step 8 have diverse grid resolutions (Table 1) and were first harmonized to the same resolution as of the dominant and aggregated crop layers of the USGS/PNSP-MRF data in step 6; remapping of grid cells values were conducted by various interpolation methods (depending on the variable to be resized) implemented using the Matlab function *resizem*. Thus, the resulting 20 homogeneous layers (one for each environmental variable) were used with the APR in 2015 relative to the 48 USA states obtained in step 7; specifically, APR values were scattered against the average value of each environmental variable *X* within each USA state for each crop type and active ingredient (Fig. 1, step 9). These scatters served to determine the “natural” correlation strength *R*_*x*_(*i*, *j*) of APR of active ingredient *i* on crop *j* against the environmental variable *X*, and the corresponding linear regression through the points. The correlation strength *R*_*x*_(*i*, *j*) was determined using the Matlab *corrcoeff* function (see natural correlation mosaics in Fig. 3 of^25,26^), while the polynomials for linear regression and inference (*APR*_*r*_) were defined as

3 $$AP{R}_{r}(i,j,\bar{X})={\alpha }_{i,j}\bar{X}+{\beta }_{i,j}$$

where $\bar{X}$ represents the generic environmental variable spatially averaged within a specific USA state and (*α*, *β*)_*i*,*j*_ are the dimensional parameters retrieved by least-squares fitting against the median APR for each pesticide *i* and crop *j* in 2015. The 95% confidence intervals *CI*(*i*, *j*, *X*) around each regression polynomial *APR*_*r*_ were calculated to determine the upper and lower APR bounds H and L, respectively. Equation (3) was assigned no value when used with the missing yield of the pastures and hays aggregated crop.

We extrapolated the APR values from the USA to the global grid using Eq. (3) with *X* in place of $\bar{X}$, where *X* is the actual value of the generic environmental variable in a specific grid cell of the remapped global grids (Fig. 1, step 10). Only grid cells in which a specific crop class existed were used. This step resulted in the raw estimate of global annual application rates *APR*_*g*_(*i*, *j*, *X*) for a specific active ingredient *i*, crop *j*, and environmental variable *X* in 2015. The overall high (H) and low (L) raw estimates $AP{R}_{g}^{* ,H}(i,j)$ and $AP{R}_{g}^{* ,L}(i,j)$ were then calculated by weight-averaging the *X*-specific estimates *APR*_*g*_(*i*, *j*, *X*) as

4a $$AP{R}_{g}^{* ,H}(i,j)=\sum _{k}{W}_{{X}_{k}}(i,j)\cdot \lfloor AP{R}_{g}(i,j,{X}_{k})+CI(i,j,{X}_{k})\rfloor $$

4b $$AP{R}_{g}^{* ,L}(i,j)=\sum _{k}{W}_{{X}_{k}}(i,j)\cdot \lfloor AP{R}_{g}(i,j,{X}_{k})-CI(i,j,{X}_{k})\rfloor $$

where the weights ${W}_{{X}_{k}}(i,j)=\left|n(i,j){R}_{{X}_{k}}(i,j)\right|/\sum _{k}\left|n(i,j){R}_{{X}_{k}}(i,j)\right|$ are relative to the extrapolation from each environmental variable *X* and their sum $\sum _{k}{W}_{{X}_{k}}(i,j)$ equals 1, and *n*(*i*, *j*) is the number of points used for polynomials fitting relative to the total number of available points (i.e., the 48 USA states). This weighting method implies that the environmental variables that were highly correlated with APR across the USA states and resulted from more available points in the USGS/PNSN database had greater weight on the global estimate as compared to the other environmental variables. In particular, we used Eq. (4) only with the 5 environmental variables with the greatest strength |*R*_*x*_(*i*, *j*)|, while the remaining variables were neglected. The *n*(*i*, *j*) and *R*_*x*_(*i*, *j*) values used in Eq. (4) were tracked to assess data quality (see “Data quality tracking and gridded quality index maps” in “Technical Validation”). The overall method described above for spatial inference of application rates was tested for robustness and statistical quality as detailed in “Validation of spatial inference” of “Technical Validation”, while full list of polynomials coefficients and goodness-of-fit metrics are distributed in^25,26^.

### Governance conditioning of global estimates (Steps 11 to 12)

The raw H and L global estimates $AP{R}_{g}^{* }(i,j)$ in Eq. (4) do not yet include specific conditions imposed by national authorities and regulations such as active ingredients that are not approved for use or banned. The raw H and L estimates were therefore conditioned (along path C1 in Fig. 1) to regulations enacted locally by combining the European Commission database on pesticides, which reports bans on active ingredients within the EU28 as of September 2016 and May 2019^27^, and the global database maintained by the Pesticide Action Network that reports bans on more than 700 active ingredients for more than 80 countries as of April 2017 and May 2019^47^. The two databases were first harmonized (Fig. 1, step 11) and were next used with a georeferenced map of countries from^48^ to revise the H and L $AP{R}_{g}^{* }(i,j)$ estimates for specific active ingredients banned on any of the dominant or aggregated crops (Fig. 1, step 12). Here, $AP{R}_{g}^{* }(i,j)$ was set to null in countries that apply a ban; the most recent known ban was assumed to last until 2025. Note that the European Community has multiple approval levels. Active ingredients approved by the European Commission also require approval by the member country before they can be used in that country, while those not approved by the Commission can be used by EU28 member countries under particular circumstances. Approval can be periodically reviewed, and hence the status of an active ingredient can change when it is not banned. For this reason, we aggregated banned and not approved (and therefore not used) substances into one class called and shown in our PEST-CHEMGRIDS release as B/NA.

### Biotechnology conditioning of global estimates (Step 13)

Biotechnologies implemented to induce resistance such as in Genetically Modified (GM) crops against specific active ingredients are explicitly accounted for in PEST-CHEMGRIDS. We retrieved the database of the International Service for the Acquisition of Agri-Biotech Applications (ISAAA), which lists 44 countries that approve pesticide-resistant GM crops as of 2018. A total of 29 crops are listed as GM in the ISAAA database, including five of the six dominant crops (corn, soyabean, wheat, cotton and alfalfa) and some of the aggregated crops used in PEST-CHEMGRIDS. We excluded the aggregated GM crops from further analysis because they cumulatively accounted for only a minor fraction of the 175 crops available to us from the MRF database. For the selected dominant GM crops, we tagged the most used active ingredients (glyphosate, glufosinate, 2,4-D, dicamba, isoxaflutole, and mesotrione) and the countries that allow both cultivation of pesticide-resistant GM crops and the use of those active ingredients (Table 4). While agronomic practices may differ for the specific ingredient used on any of the dominant GM crops, we assumed that the application rate of GM crop-specific active ingredients in the USA is the upper bound, while the APR on the corresponding non-GM crops in other countries was taken as 30% of the one in the USA. Use of the APR in the USA as the upper bound was justified by the fact that the USA does not apply restrictions or bans to the selected GM crop-specific active ingredients. In addition, countries that do not allow for GM crops but do not have a ban on GM crop-specific active ingredients can, by a matter of fact, use that active ingredient, but that likely occurs at lower application rates. For example, glyphosate-resistant GM corn in the EU is not allowed for feeding purpose but there is no ban on glyphosate, which can be used with no or minor restrictions; hence, the amount used was presumed to be substantially less than in countries where glyphosate-resistant GM corn is allowed. This approach is corroborated by a reanalysis of data in^49^ showing a generally higher applied glyphosate mass on glyphosate-resistant cotton, corn and soyabean as compared to the corresponding traditional crops in the USA over the period 1998 to 2009. Finally, active ingredients used with GM crops were excluded if a ban was lifted in a specific country even if GM crops are permitted. These conditioning in our estimates were implemented in Fig. 1, step 13 along path C2 of the workflow

**Table 4:** List of GM crops and country approvals.

GM crop	Resistance	Approving countries
Alfalfa	Glyphosate	Argentina, Canada, Japan, Mexico, USA
Cotton	Glyphosate	Argentina, Australia, Brazil, Colombia, Costa Rica, Japan, Mexico, Paraguay, South Africa, USA
	Glufosinate	Argentina, Australia, Brazil, Colombia, Costa Rica, Japan, Mexico, USA
	2,4-D	Brazil, Costa Rica, Japan, USA
	Dicamba	Australia, Brazil, Costa Rica, Japan, USA
	Isoxaflutole	USA
Corn	Glyphosate	Argentina, Brazil, Canada, Chile, Colombia, Cuba, Egypt, Honduras, Japan, Pakistan, Paraguay, Philippines, South Africa, USA, Uruguay, Viet Nam
	Glufosinate	Argentina, Brazil, Canada, Colombia, Honduras, Japan, Pakistan, Panama, Paraguay, Philippines, South Africa, USA, Uruguay, Viet Nam
	2,4-D	Argentina, Brazil, Canada, Japan, USA
	Dicamba	Brazil, Canada, USA
Rice	Glufosinate	USA
Soyabean	Glyphosate	Argentina, Bolivia, Brazil, Canada, Chile, Costa Rica, Japan, Mexico, Paraguay, South Africa, USA, Uruguay
	Glufosinate	Argentina, Brazil, Canada, Japan, USA, Uruguay
	2,4-D	Argentina, Brazil, Canada, Japan, USA
	Dicamba	Brazil, Canada
	Isoxaflutole	Argentina, Brazil, Canada, Japan, USA
	Mesotrione	Argentina, Canada, Japan, USA

This is an extract of the database of dominant GM crops and active ingredients resistance in^60^ used by various countries and accounted for in PEST-CHEMGRIDS for biotechnology conditioning implemented in Fig. 1, step 13 along path C2.

### Global conditioning against FAOSTAT pesticide records (Step 14)

The last step to estimate the global pesticide application rates was conducted to correct biases introduced by the methods described above using the FAOSTAT pesticide database^1^, which reports the cumulative pesticides mass and the mass of pesticides grouped by herbicides, insecticides, and the lumped fungicides and bactericides applied country-wide from 1990 to 2016 (Fig. 1, step 14). To correct our raw estimates in 2015 from step 13, the mass in each country was calculated by integrating the median $AP{R}_{g}^{* }(i,j)$ for ingredient *i* on crop *j* in 2015. This country-specific cumulative mass, *M*_*c*_, was next compared to the values *M*_*c*,*FAO*_ in the FAOSTAT and the ratio *R*_*c*_ = *M*_*c*,*FAO*_/*M*_*c*_ was calculated. The closer is *R*_*c*_ to 1, the closer our estimate to the FAOSTAT data in a specific country. Several estimates were fairly close to the FAOSTAT data even before implementing this conditioning procedure, but a number of corrections were to be implemented. Hence, *APR*_*c*_ in all countries other than the USA was conditioned to *M*_*c*,*FAO*_ in 2015 as (Fig. 1, step 14 path C3)

5a $$AP{R}_{g,c}^{H}(i,j)=AP{R}_{g,c}^{* ,H}(i,j)\cdot {\rm{\min }}\left[{F}_{M}\cdot {R}_{c},\frac{AP{R}_{USA}^{* ,H}(i,j)}{AP{R}_{USA}^{* ,L}(i,j)}\right]$$

5b $$AP{R}_{g,c}^{L}(i,j)=AP{R}_{g,c}^{* ,L}(i,j)\cdot {\rm{\min }}\left[{F}_{M}\cdot {R}_{c},\frac{AP{R}_{USA}^{* ,H}(i,j)}{AP{R}_{USA}^{* ,L}(i,j)}\right]$$

where *F*_*M*_ = 0.842 is the correction factor introduced in Section “Selection of active ingredients samples” to account for the mass deficit relative to the total mass of pesticides in the USA in 2015, while the second term within square parenthesis relative to the USA was used as a limiting factor to prevent outliers of a particular ingredient when the correction to be implemented was substantial. This conditioning was not applied to the application rates in the USA because they were determined from the USGS/PNSN-MCR database and matched relatively well the FAOSTAT.

During this conditioning, we tracked the number of countries for which an estimate of the total pesticide mass was available in the FAOSTAT database; it was noted that the FAOSTAT reports data for about 160 countries. Hence, the ratio *R*_*c*_ for missing countries in 2015 was determined as the average *R*_*c*_ of the neighbouring countries based on the assumption that geographic proximity is a measure of similarities in agricultural, environmental, and socio-economic variables^50^. Figure 3a shows the corrected high (H) and low (L) APR estimates for individual countries as compared to the FAOSTAT data in 2015, while Fig. 3b shows the resulting global APR projections (for the median) as compared to the FAOSTAT historical data from 1992 to 2016 calculated for all countries and the countries reported in the FAOSTAT database for comparison.

The conditioning implemented to APR in this step is the result of comparison with the total pesticide use in a country; however, the FAOSTAT also provides disaggregated data for herbicides, insecticides, and bactericides and fungicides, which were used for validation and data quality tracking described in “Technical Validation”.

### Projections of global estimates (Step 14)

Finally, the projections of APR from 2015 to 2025 were obtained by applying the prefactors *F* in Eq. (4) to scale the raw global estimates of Eq. (5) in 2015 according to the trends in pesticide mass historically observed in the USA and calculated in step 5. The projections were estimated as

6a $$AP{R}_{g}^{H}(i,j,{t}_{p})=F(i,j,{t}_{p})\cdot AP{R}_{g}^{H}(i,j)$$

6b $$AP{R}_{g}^{L}(i,j,{t}_{p})=F(i,j,{t}_{p})\cdot AP{R}_{g}^{L}(i,j)$$

for *t*_*p*_ ranging between 2015 and 2025, and with $AP{R}_{g}^{H}(i,j)$ and $AP{R}_{g}^{L}(i,j)$ from Eq. (5).

**Fig. 4:**
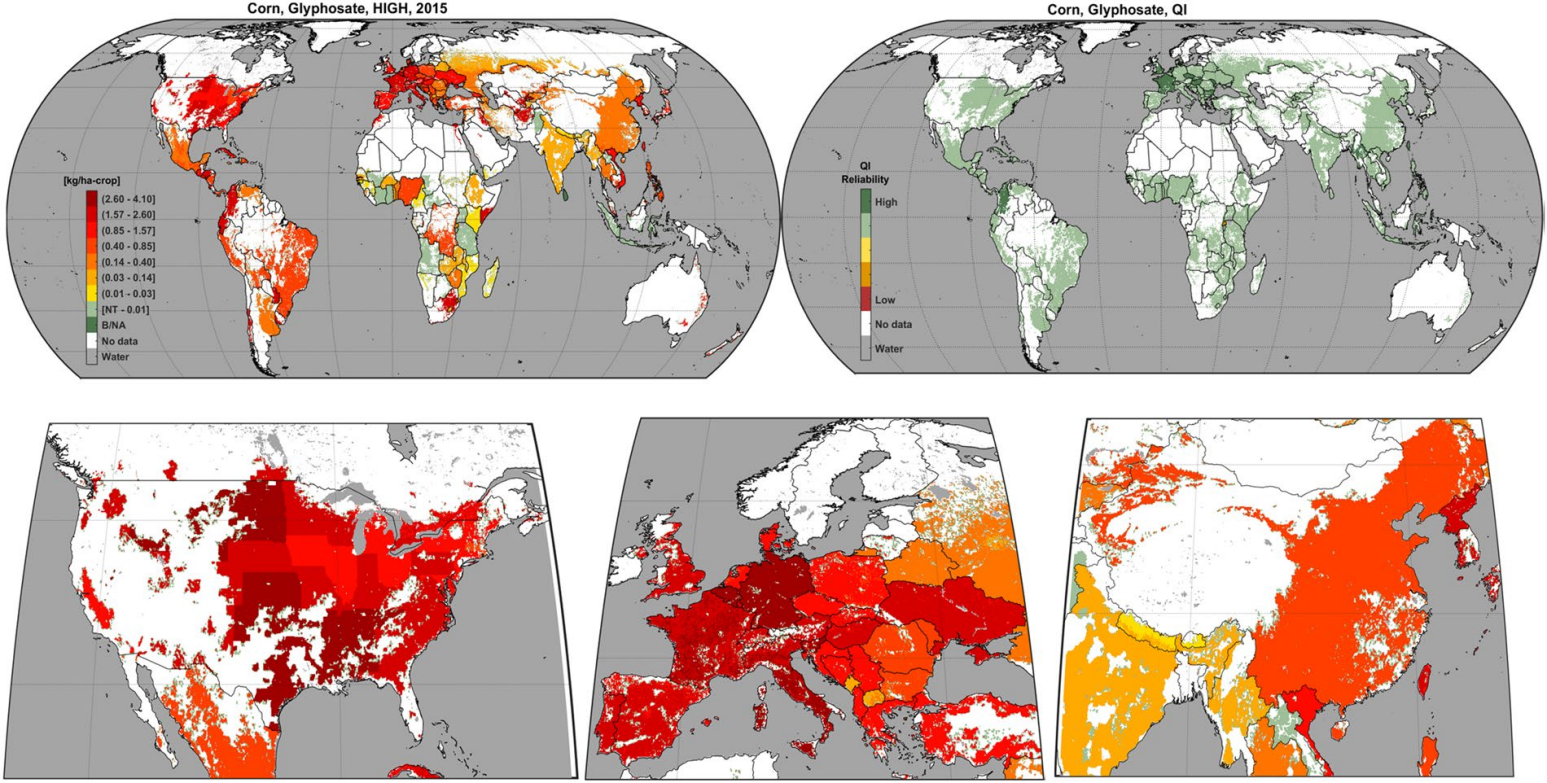
Examples of global gridded application rate and quality index maps. The top two panels show the high (HIGH) estimate in 2015 for the annual application rate of glyphosate on corn globally gridded and the corresponding quality index QI map. Panes in the second row show regional application rates.

These global estimates ultimately describe the subnational distribution of the annual application rate of the 20 most used pesticides active ingredients on 6 dominant and 4 aggregated crops from 2015 to 2025. The PEST-CHEMGRIDS release also includes gridded maps of the quality index *QI* describing the reliability of estimates in each grid cell for all ingredients and crops, which is described in “Technical Validation”. Examples of maps for estimates relative to the most used active ingredients on corn (glyphosate) and its corresponding quality index *QI* map are provided in Fig. 4. The full PEST-CHEMGRIDS data set is accessible in^25,26^, while the full list of crop-specific active ingredients maps is provided in Table 5.

**Table 5:** List of APR and QI maps in the PEST-CHEMGRIDS release.

Crop class	Top 20 crop-specific active ingredients
Corn	glyphosate (HBC), atrazine (HBC), acetochlor (HBC), metolachlor(-s) (HBC), 2,4-d (HBC, PGR), propargite (ACA), simazine (HBC), dimethenamid(-p) (HBC), mesotrione (HBC), dicamba (HBC), paraquat (HBC), pendimethalin (HBC), terbufos (INS), chlorpyrifos (ACA, INS), alachlor (HBC), clopyralid (HBC), glufosinate (HBC), pyraclostrobin (FUN, PGR), isoxaflutole (HBC), azoxystrobin (FUN)
Soyabean	glyphosate (HBC), metolachlor(-s) (HBC), 2,4-d (HBC, PGR), fomesafen (HBC), acetochlor (HBC), glufosinate (HBC), pendimethalin (HBC), metribuzin (HBC), sulfentrazone (HBC), paraquat (HBC), trifluralin (HBC), dimethenamid(-p) (HBC), acephate (INS), dicamba (HBC), chlorpyrifos (ACA, INS), clethodim (HBC), acifluorfen (HBC), flumioxazin (HBC), pyraclostrobin (FUN, PGR), pyroxasulfone (HBC)
Wheat	glyphosate (HBC), 2,4-d (HBC, PGR), mcpa (HBC), bromoxynil (HBC), propiconazole (FUN), tebuconazole (FUN), fluroxypyr (HBC), paraquat (HBC), dicamba (HBC), clopyralid (HBC), chlorpyrifos (ACA, INS), prothioconazole (FUN), azoxystrobin (FUN), atrazine (HBC), dimethoate (ACA, INS), tri-allate (HBC), pyraclostrobin (FUN, PGR), thiophanate-methyl (FUN), pinoxaden (HBC), metconazole (FUN, PGR)
Cotton	glyphosate (HBC), dichloropropene (HBC, NEM), trifluralin (HBC), acetochlor (HBC), glufosinate (HBC), metolachlor(-s) (HBC), paraquat (HBC), pendimethalin (HBC), acephate (INS), diuron (HBC), prometryn (HBC), msma (HBC), dicrotophos (ACA, INS), 2,4-d (HBC, PGR), fluometuron (HBC), fomesafen (HBC), dicamba (HBC), bifenthrin (ACA, INS), chlorpyrifos (ACA, INS), imidacloprid (INS)
Rice	propanil (HBC), thiobencarb (HBC), glyphosate (HBC), copper sulfate (FUN), clomazone (HBC), pendimethalin (HBC), quinclorac (HBC), propiconazole (FUN), azoxystrobin (FUN), imazethapyr (HBC, PGR), 2,4-d (HBC, PGR), triclopyr (HBC), cyhalofop (HBC), trifloxystrobin (FUN), cyhalothrin-lambda (INS), halosulfuron (HBC), acifluorfen (HBC), clothianidin (INS), bentazone (HBC), saflufenacil (HBC)
Alfalfa	glyphosate (HBC), pendimethalin (HBC), trifluralin (HBC), chlorpyrifos (ACA, INS), diuron (HBC), 2,4-db (HBC), malathion (ACA, INS), metribuzin (HBC), hexazinone (HBC), carbaryl (INS, PGR), dimethoate (ACA, INS), 2,4-d (HBC, PGR), eptc (HBC), paraquat (HBC), clethodim (HBC), phosmet (INS), sethoxydim (HBC), bromoxynil (HBC), cyhalothrin-lambda (INS), indoxacarb (INS)
VegFru	metam (FUN, HBC, INS, NEM), dichloropropene (HBC, NEM), metam potassium (FUN, HBC, INS, NEM), chloropicrin (NEM), chlorothalonil (FUN), glyphosate (HBC), mancozeb (FUN), eptc (HBC), metolachlor(-s) (HBC), petroleum oil (ACA, FUN, HBC, INS), bentazone (HBC), pendimethalin (HBC), ethoprophos (INS, NEM), bacillus amyloliquifacien (FUN), copper hydroxide (FUN), bensulide (HBC), captan (FUN), methyl bromide (FUN, HBC, INS, NEM), thiophanate-methyl (FUN), ethalfluralin (HBC)
OrcGra	petroleum oil (ACA, FUN, HBC, INS), glyphosate (HBC), dichloropropene (HBC, NEM), copper hydroxide (FUN), calcium polysulfide (ACA, FUN, INS), captan (FUN), mancozeb (FUN), pendimethalin (HBC), chlorpyrifos (ACA, INS), paraquat (HBC), ziram (FUN, REP), chlorothalonil (FUN), copper sulfate tribasic (FUN), glufosinate (HBC), copper sulfate (FUN), diuron (HBC), chloropicrin (NEM), oxyfluorfen (HBC), 2,4-d (HBC, PGR), methyl bromide (FUN, HBC, INS, NEM)
PasHay	glyphosate (HBC), 2,4-d (HBC, PGR), dicamba (HBC), atrazine (HBC), triclopyr (HBC), picloram (HBC), mcpa (HBC), paraquat (HBC), aminopyralid (HBC), 2,4-db (HBC), dichlorprop (HBC), imazapyr (HBC), fluroxypyr (HBC), glufosinate (HBC), clopyralid (HBC), metribuzin (HBC), metolachlor(-s) (HBC), diuron (HBC), clethodim (HBC), metsulfuron (HBC)
Other	glyphosate (HBC), atrazine (HBC), dichloropropene (HBC, NEM), metolachlor(-s) (HBC), chlorothalonil (FUN), chloropicrin (NEM), bacillus amyloliquifacien (FUN), 2,4-d (HBC, PGR), pendimethalin (HBC), metam (FUN, HBC, INS, NEM), acetochlor (HBC), metribuzin (HBC), dicamba (HBC), phorate (INS), chlorpyrifos (ACA, INS), flutolanil (FUN), paraquat (HBC), propazine (HBC), dimethenamid(-p) (HBC), bromoxynil (HBC)

For each crop class in column 1, maps are produced for every active ingredient listed in column 2 (list is in decreasing order by application rate) for years 2015, 2020 and 2025, and for both high (H) and low (L) estimates. Maps of data quality are produced for each crop and active ingredient as well. Files are equally stored in Portable Network Graphics (.PNG), Tagged Image File Format (.TIFF/.TIF), and NetCDF4 (.NC) formats. HBC, INS, FUN, ACA, PGR, NEM, and REP stand for ‘herbicide’, ‘insecticide’, ‘fungicide’, ‘acaricide’, ‘plant growth regulator’, ‘nematicide’, and ‘repellent’, respectively.

### A global outlook

**Fig. 5:**
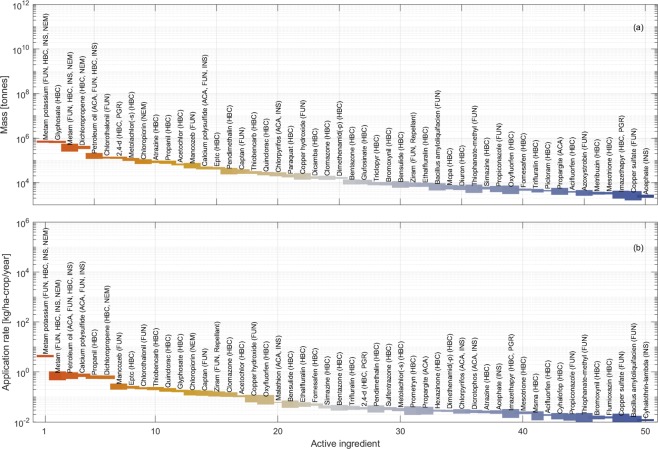
Global outlook. The mass (**a**) and application rate (**b**) are represented for the top 50 active ingredients globally. Ingredients are sorted in decreasing order of global applied mass and application rate. HBC, INS, FUN, ACA, PGR, NEM, and REP stand for ‘herbicide’, ‘insecticide’, ‘fungicide’, ‘acaricide’, ‘plant growth regulator’, ‘nematicide’, and ‘repellent’, respectively.

We summarise the estimated mass and application rates of 50 of the 95 selected active ingredients used globally (Fig. 5). With reference to the mass, the most used herbicides resulted to be glyphosate and metam potassium (about 700,000 tonnes per year), metam and dichloropropene (about 450,000 tonnes per year) and 2,4-D (about 150,000 tonnes per year). The most used insecticides are metam potassium and metam, calcium polysulfide (about 50,000 tonnes per year) and chlorpyrifos (about 20,000 tonnes per year). Finally, the most used fungicides are metam potassium, petroleum oil (about 150,000 tonnes per year), and chlorothalonil (about 120,000 tonnes per year).

## Data Records

The PEST-CHEMGRIDS data release has a global extent with bounding box 180°E–180°W; 56°S–84°N at a resolution of 5 arc-min by 5 arc-min (about 10 km by 10 km at the equator) in standard WGS84 coordinates, corresponding to matrices of 1681 (S-N) by 4306 (E-W) pixels. PEST-CHEMGRIDS stored in^25,26^ is organized in three compressed folders, each collecting files of the same maps in a different format, namely Portable Network Graphics (.PNG), Tagged Image File Format (.TIFF/.TIF), and NetCDF4 (.NC) to facilitate distribution and usability. The full list of maps is provided in Table 5 with the corresponding active ingredient and crop class, and with some details on formats. Intermediate data such as those prior to estimate conditioning as per workflow in Fig. 1 are available upon request. All source data are detailed in Table 1 and are accessible from the original repositories.

## Technical Validation

The technical validation of the PEST-CHEMGRIDS data release is structured into three levels: (i) benchmarking of source data, (ii) estimates conditioning and validation against independent data, and (iii) data quality tracking across key implementation steps and data reliability calculations. The validation levels are described in detail below.

### Source data benchmarking

**Fig. 6:**
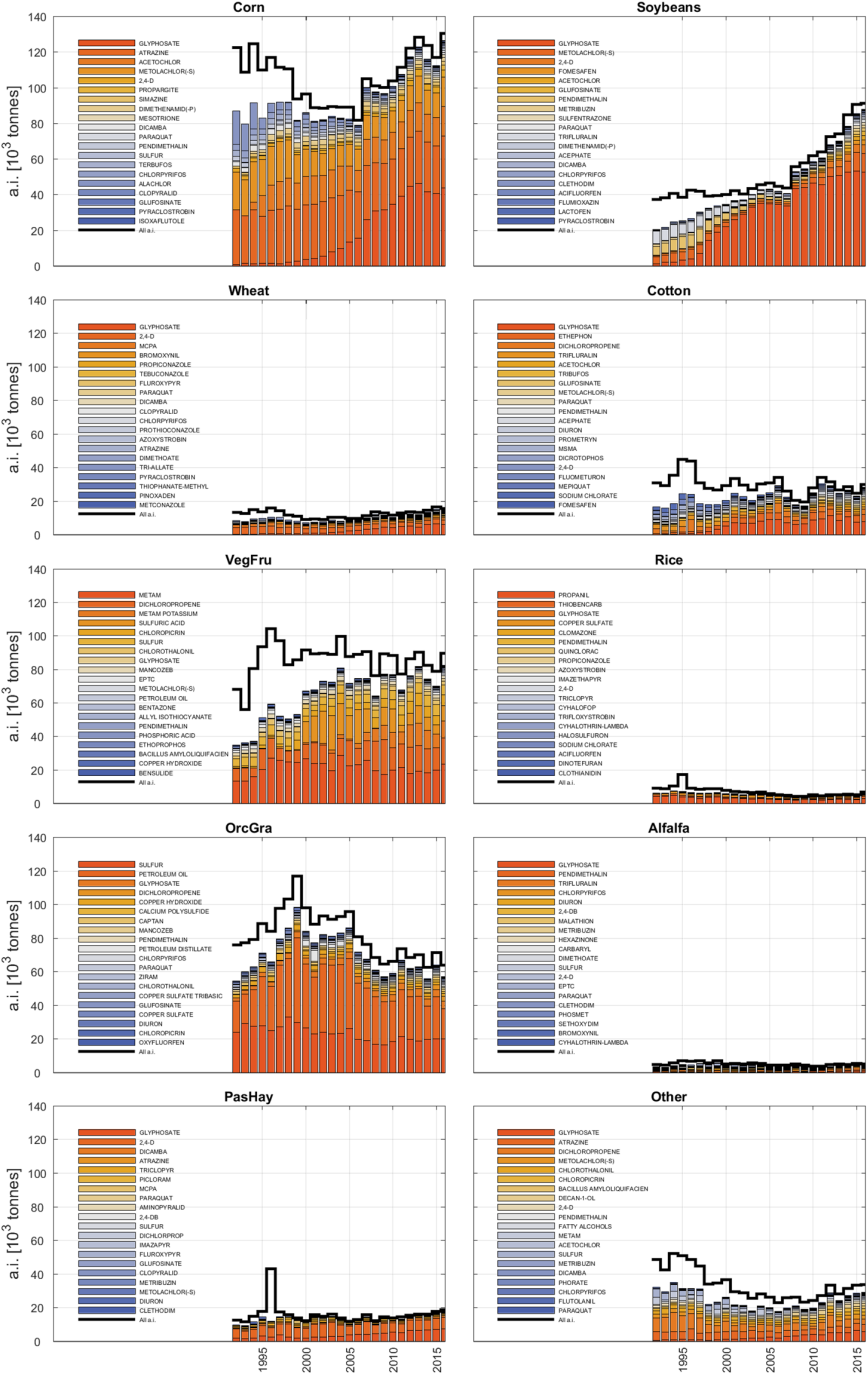
Patterns in pesticide use in the USA. Cumulative mass of all (black line) and the 20 most used crop-specific active ingredients (colour bars) in the USA from 1992 to 2016. Data are redrawn from the USGS/PNSP database in^22^. a.i. refers to active ingredients.

The USGS/PNSP data were reanalysed via step 1 to 2 to retrieve statistical information of the top 20 crop-specific active ingredients and to detect patterns that could bias our estimates. Selected active ingredients ranked by the mass applied in 2015 (Fig. 6) show that they represented the greatest fraction (up to about 84.2%) of the total applied in the USA, but also show that these have had less relevance in previous years such as in the evident case of corn and soybean. For other crops, the most used 20 ingredients in 2015 retained a relevant presence also in previous years such as in the case of rice and alfalfa. Hence, some patterns in pesticide use have undergone substantial changes over the past 25 years; this justifies the choice to calculate APR projections based on the most recent pesticide use distributions, bearing in mind that this may be subject to changes over the 2015–2025 period.

**Fig. 7:**
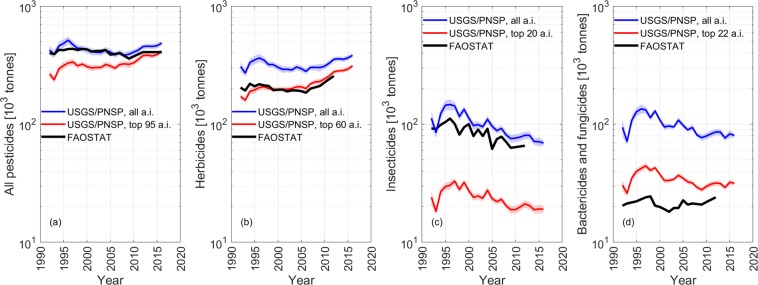
Reanalysis and benchmarking of USGS/PNSP against FAOSTAT. (**a**) Represents the mass in all and the 95 selected pesticides; (**b**–**d**) refer to the mass in all and the selected 60 “herbicides”, 20 “insecticides and seed treatments”, and 22 “bactericides and fungicides”, respectively. The selected active ingredients (a.i.) are the cumulative of the top 20 crop-specific ingredients grouped by pesticide class as in the FAOSTAT database.

All and the top 20 crop-specific active ingredients in the USA were classified in step 2 according to the EU28 pesticide classification database^27^. Note that the 20 crop-specific ingredients cumulatively resulted in 95 most used ingredients across all dominant and aggregated crops. These were benchmarked against the FAOSTAT pesticide database (Fig. 1,B1). That is, the class-specific cumulative “high” and “low” USGS/PNSP estimates matched the FAOSTAT data well with only a minor overestimate for all pesticides (Fig. 7a) and pesticides classes (Fig. 7b–d). “Insecticides” and “fungicides and bactericides” were likely overestimated (blue lines) because we included seed treatments, which were not included in the FAOSTAT database. The similar masses in the last two pesticides classes may be due to the classification counting. For example, mass of active ingredients falling in multiple classes were counted multiple times and divided by the number of belonging classes. Overall, the mismatch in the “insecticides” and in the “bactericides and fungicides” classes was more than one order of magnitude smaller than the total pesticide mass and, hence, was considered to introduce only minor errors. These errors were not corrected because source data are from two independent authorities.

### Validation of historical trends and projection prefactors

We tested polynomials of order 1 to 3 on historical USGS/PNSP data to achieve projections from 2015 to 2025. Polynomials of order greater than 1 resulted in better fit to historical data of individual active ingredients on specific crops but projections suffered from unrealistic, steep changes in a few years ahead of 2015; upon appreciation that first-order polynomials captured relatively well the historical trends over 25 years in the USA for most ingredients and crops (see dashed lines in Fig. 2 and^25,26^ for comparisons with higher-order polynomials) and with partial consideration of the actual goodness-of-fit of higher-order polynomials, first-order polynomials were used for projections. Note that while the projection of one active ingredient on one crop was the result of only one linear approximation, the projected global use of pesticides or pesticide classes combine multiple linear approximations and can result in segmented (piecewise) trends.

### Validation of spatial inference methods

We tested monovariate and multivariate polynomials ranging from order 1 to 3 and from order 1 to 2, respectively, for spatial inference of APR values from the USA to globally. We used a linear combination of monovariate polynomials (function *polyfit* in Matlab) with weights as described in Section “Global spatial inference of application rates”, that is, weights were proportional to the “natural” correlation between application rates and individual independent variables. We tested multivariate polynomials with and without interactions (function *fitlm* in Matlab), that is, including and excluding combinatorial products of independent variables, respectively, but we did not use linear combinations because this method only returns one polynomial. Spatial inference tests were conducted on gridded maps of applied atrazine on corn at sub-county level within the USA in year 1997 available from^22^, which includes 35,000 grid cells. Bin averages were first calculated to reduce data dispersion; we then divided the resulting points for the application rates and the 20 environmental variables into randomized calibration and validation sets. We tested different calibration and validation set sizes. We then applied the above methods and we calculated the goodness-of-fit (R and NRMSE) on the validation sets. Excluding the pure quadratic multivariate polynomial fitting (Table 6), all other methods were nearly equivalent in terms of goodness-of-fit. Focusing only on the 0.07 fraction of points for calibration (this is the case most similar to our global spatial inference), the rank in the right most column of Table 6 ranged between about 30 and 40 (minimum is 0 for best, above 100 is poor). Although the weighted linear combination of monovariate second-order polynomials performed relatively well, we ultimately used monovariate first-order polynomials after considering that: (i) high-order polynomials are known to produce better fit on tests points and introduce distortions to extrapolation points far from the calibration points, which could be the case in a number of combinations of active ingredients and crops in PEST-CHEMGRIDS beyond these tests; and (ii) the chosen method is simple to implement even in computational environment different than the one we used (Mathworks Matlab) by following Eqs (3 and 4). The full list of polynomials coefficients and quality of regression is provided in^25,26^.

**Table 6:** Statistics on quality of spatial inference methods.

Method	Order of polynomials	Fraction of data for calibration	Number of points for calibration	Number of points for validation	Correlation coefficient R	Normal. Root mean square error NRMSE (%)	Rank (1 − |R|) * 100 + NRMSE Minimum = best
Monovariate with weighted linear combination	1 (linear)	0.07	49	656	0.701	10.33	40.2
		0.2	141	564	0.674	10.80	43.4
		0.5	352	353	0.649	12.90	48.0
	2 (quadratic)	0.07	49	656	0.775	9.10	31.6
		0.2	141	564	0.709	10.31	39.4
		0.5	352	353	0.689	12.34	43.4
	3 (cubic)	0.07	49	656	0.774	9.16	31.8
		0.2	141	564	0.716	10.26	38.7
		0.5	352	353	0.696	12.24	42.6
Multivariate without interaction products	1 (linear)	0.07	49	656	0.719	10.74	38.8
		0.2	141	564	0.752	9.25	34.1
		0.5	352	353	0.772	10.48	33.3
	2 (quadratic)	0.07	49	656	0.437	13.65	70.0
		0.2	141	564	−0.173	15.51	98.2
		0.5	352	353	−0.177	17.66	100.0
Multivariate with interaction products	1 (linear)	0.07	49	656	0.719	10.95	39.1
		0.2	141	564	0.760	9.39	33.4
		0.5	352	353	0.842	8.83	24.6
	2 (quadratic)	0.07	49	656	0.741	10.88	36.8
		0.2	141	564	0.740	9.51	35.5
		0.5	352	353	0.776	10.35	32.8

Monovariate polynomials were calculated using the Matlab function *polyfit*, while multivariate polynomials were calculated with the Matlab function *fitlm*.

### Validation of conditioned estimates against FAOSTAT aggregated pesticide classes

Statistical inference first (Fig. 1, step 10) and the following conditioning of global estimates to country-specific governances, biotechnologies, and records of total pesticide application mass (Fig. 1, steps 12 to 14) do not ensure alone that the estimates are of enough high quality to be usable by third parties. The ultimate validation of estimates was conducted on independent data from the FAOSTAT pesticide database. As mentioned earlier, the FAOSTAT includes the country-specific cumulative mass of “herbicides”, “insecticides”, and lumped “bactericides and fungicides” from 1990 to 2016. These aggregated pesticide classes do not specify the exact active ingredients; yet, comparison with our selection of the top 20 most used crop-specific ingredients in the USA in 2015 (i.e., 84.2% of the total pesticide mass) showed that the ingredients were distributed nearly as in the FAOSTAT pesticide aggregated classes over time (Fig. 6). Validation, therefore, was conducted along pathway B2 in Fig. 1 by integrating the H and L APR estimates in all countries using the surface area of dominant and aggregated crops, and tracking the class of each active ingredient. The mass of all ingredients within the same class as in the FAOSTAT was therefore aggregated correspondingly. To calculate country integrals, we used the global country borders map available in^48^.

**Fig. 8:**
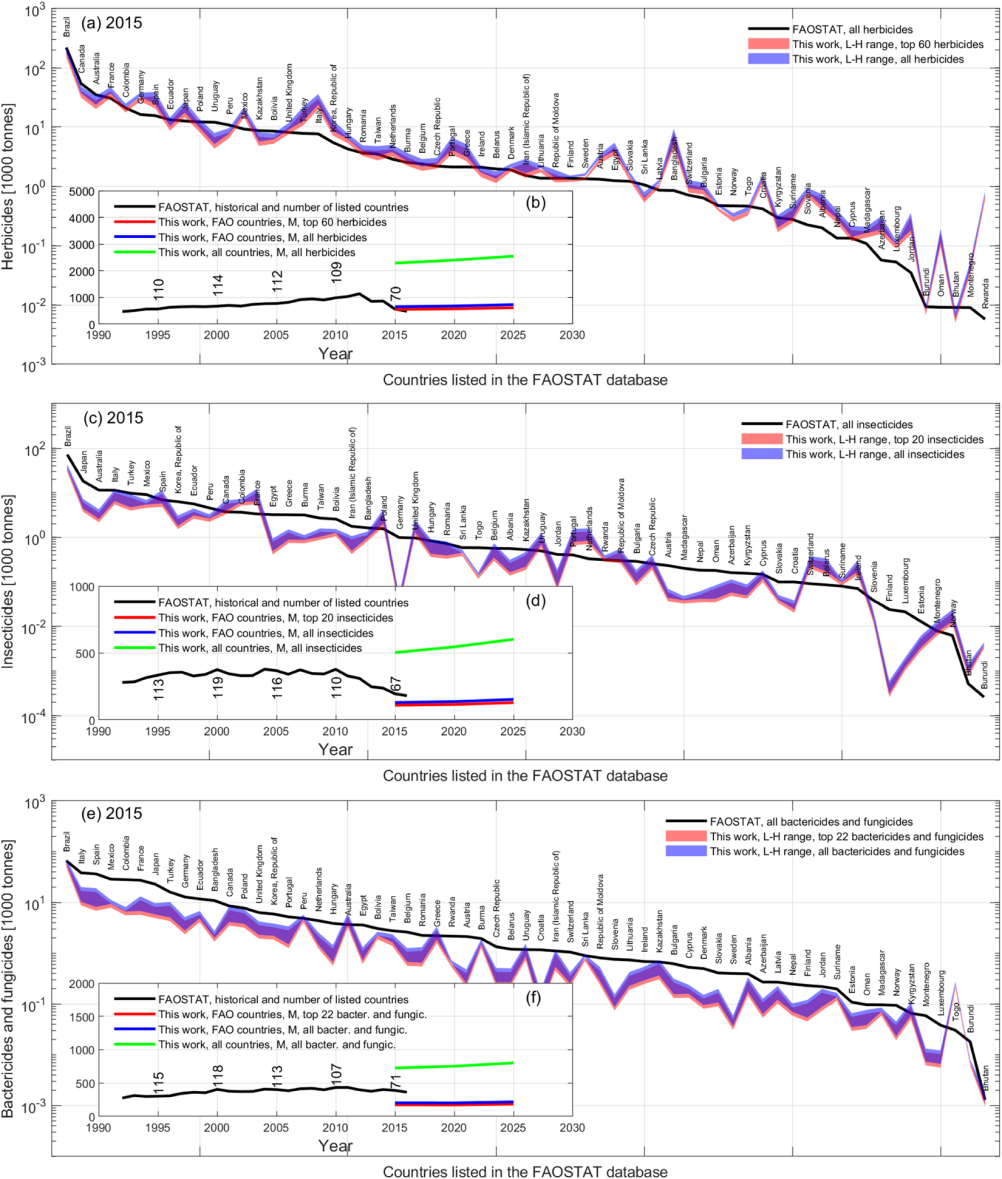
Validation of estimates. The main panels (a–c) represent the country-specific cumulative mass of active ingredients reported in the FAOSTAT pesticide database and the PEST-CHEMGRIDS estimates for “herbicides”, “insecticides”, and “bactericides and fungicides”, respectively, in 2015. Estimates for all a.i. in blue were obtained using the correction factor *F*_*M*_ = 0.842. Inset panels (b,d,f) represent the global cumulative mass of the corresponding projections from 2015 to 2025 relative to the countries listed in the FAOSTAT and all countries. Ideally, the blue line should be as closer as possible to the FAOSTAT historical data. Panel (c) excludes Sweden, Denmark, Latvia and Lithuania because none or only a few insecticides selected in PEST-CHEMGRIDS are allowed in those countries, and thus comparison is not possible even if they are included in the FAOSTAT database.

Our estimates of the herbicide mass used in each country available in the FAOSTAT in 2015 matched the FAOSTAT data well (Fig. 8a), with the country-specific average error of the median estimate relative to FAOSTAT being about 2.5. The relative error for a country was calculated as |*X*_EST_ − *X*_FAOSTAT_|/*X*_FOASTAT_, where *X*_EST_ is the estimated value for a country and *X*_FAOSTAT_ is the FAOSTAT value for the same country, which was next averaged over the FAOSTAT countries to retrieve the average error. The global herbicide estimate was also close to the FAOSTAT data (Fig. 8b, red line). Note, however, that the number of countries in the FAOSTAT varied over the years and showed a sensitive decline since 2010 down to 70 countries in 2015, meaning that the FAOSTAT underestimates the actual total global herbicide mass. Our estimate for all countries (including the UN193 countries as of 2019) suggests that the global herbicide mass is nearly 3 times that of the FAOSTAT (Fig. 8b, green line).

We used a similar approach to validate our estimates against the country-specific and global insecticides mass in 2015. Relative to the countries of the FAOSTAT, the validation error of the median estimate was 0.07 averaged across the countries (Fig. 8c), with the total insecticide mass slightly underestimating that of the 67 countries in the FAOSTAT (Fig. 8d). Our global estimate over all countries suggests larger values but we could not conclude if these are in line with the FAOSTAT records because only 9 out of 67 countries are associated with data on insecticides used for seed treatment in 2015.

Finally, an analogous validation of country-specific and global “bactericides and fungicides” applications in 2015 shows a relative average error of 0.64 (Fig. 8e) and a slightly underestimated global mass, respectively (Fig. 8f). In contrast to the reported 59 countries in the FAOSTAT, we overall estimate a global mass about 3 times greater than in the FAOSTAT, but we recall that only 13 out of 71 countries were associated with data on “bactericides and fungicides” used for seed treatments in 2015.

### Validations against independent pesticide databases by active ingredient

**Fig. 9:**
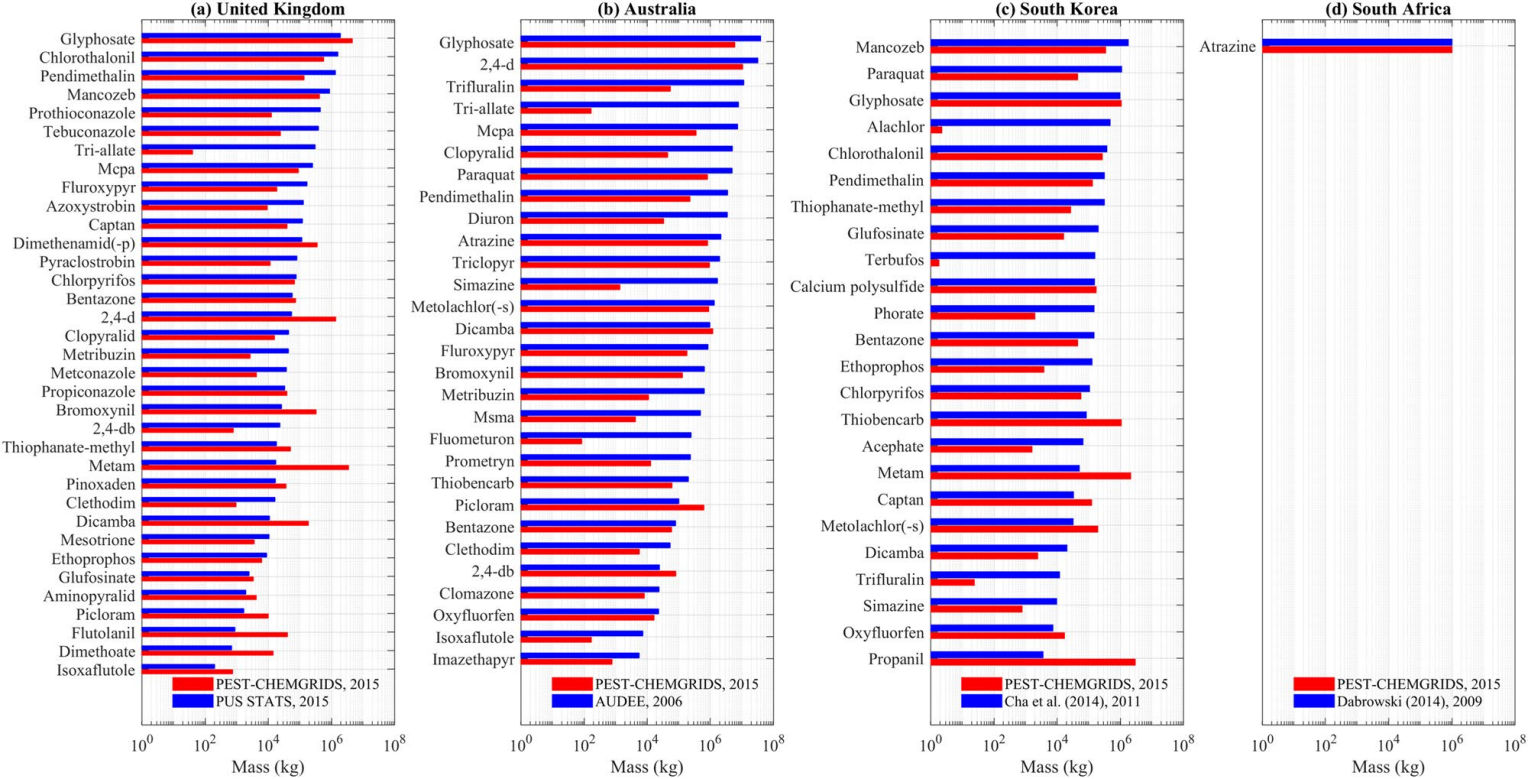
Validation of estimates against data in the United Kingdom, Australia, South Korea, and South Africa. (**a**) masses of active ingredients applied in the United Kingdom in the year 2015 were sourced from PUS STATS^51^, (**b**) masses of active ingredients applied in Australia relative to the year 2006 were obtained from AUDEE^52^, (**c**) masses of active ingredients applied in South Korea relative to the year 2011 were sourced from^53^, and (**d**) atrazine mass applied in South Africa in the year 2009 was reported in^54^.

Along with the validation against FAOSTAT, we compared the PEST-CHEMGRIDS estimates against independent pesticide databases or literature reporting the mass of individual active ingredients applied in a specific country in a given year. We used data of 35 active ingredients mass applied in the United Kingdom in 2015 reported in PUS STATS^51^, 29 active ingredients applied in Australia in 2006 reported in the Agricultural Chemical Usage Database of the Australian Department of the Environmental and Energy (AUDEE)^52^, 24 active ingredients applied in South Korea in 2011 reported in^53^, and atrazine applied in South Africa in 2009 reported in^54^. The comparison in Fig. 9 shows generally a good match between PEST-CHEMGRIDS and independent sources, though some estimates have discrepancies. The relative errors averaged over the active ingredients are 9.8 (UK), 0.98 (AU), 37.6 (SK) and 0.009 (SA). PEST-CHEMGRIDS overestimated the applied masses of 5 active ingredients in the United Kingdom by about an order of magnitude (e.g., 2,4-d, metam, dicamba, Fig. 9a) and underestimated the use of some active ingredients in Australia (e.g., trifluralin, simazine, fluometuron, Fig. 9b). Specific to South Korea, PEST-CHEMGRIDS matched relatively well the masses reported in^53^ but underestimated alachlor, terbufos, and trifluralin (Fig. 9c,d). We conclude that variability against independent records is present but overall trends are well captured.

### Validation of conditioned estimates against manufacturers’ recommendations

In addition to the above validations, we conducted a separate quality control on estimates by identifying values of application rates for active ingredients that were particularly high as compared to other ingredients, and we compared our estimates to those recommended by the manufacturers or regulatory bodies. For example, values of the fumigant metam potassium up to about 160 kg/ha-crop were found particularly high in the crop class “VegFru” but are in the range of or lower than the recommended 40 to 360 kg/ha-crop in the US EPA^55^. Similarly, chloropicrin in the “VegFru” crop class was estimated up to about 22 kg/ha-crop, which meets recommendations or is below the maximum application rates of about 350 kg/ha-crop^56^. Dichloropropene applications up to about 40 kg/ha in the “VegFru” class meet the recommended maximum application rates of about 370 kg/ha^57^, while chlorothalonil estimates below 7 kg/ha-crop in “VegFru” are substantially lower than the maximum application rate of 1400 kg/ha-crop reported in the product factsheet. In contrast, calcium polysulfide (lime sulphur) estimates up to about 25 kg/ha-crop in “OrcGra” crop class is overestimated as compared to recommended 1 to 1.2 kg/ha-crop^58^.

### Data quality tracking and gridded quality index maps

Throughout the workflow depicted in Fig. 1, we have identified crucial steps to measure the quality of our estimates. Five specific quality factors *QF* were therefore defined to cover three levels of specific data quality metrics.

*QF*_*SA*_ (Fig. 1 steps 1 to 6) quantifies the quality of the aggregation matrix for the surface area of dominant and aggregated crops used for the raw APR estimates. *QF*_*SA*_ was defined by a vector of 1 s for the dominant crops used in the USGS/PNSP database that have equivalent representation in the MRF database used for our estimates, that is, for corn, soyabean, wheat, cotton, rice and alfalfa. For all aggregated crops, *QF*_*SA*_ is calculated from the average fraction of matched crops within the two USGS/PNSP and the MRF databases. For example, the vegetables and fruits aggregated crop in the USGS/PNAS database (VegFru, Table 1) consists of a pool of 58 crops with 19 unmatched crops, hence the matched fraction is 0.673; correspondingly, the crops pool used in PEST-CHEMGRIDS consists of 12 partially matched crops out of 58 crops, hence the matched fraction is 0.793. The resulting quality factor *QF*_*SA*_ = (0.673 + 0.793)/2 = 0.733 is used for the VegFru aggregated crop. A similar procedure is used for aggregated orchards and grapes (OrcGra), and other crops (Other). For aggregated pastures and hays (PasHay), *QF*_*SA*_ is not defined as detailed earlier in section “Application rate in the USA”, hence this factor is not further accounted for on this crop class.

*QF*_*HT*_ (Fig. 1 steps 1 to 3) quantifies the quality of regression on historical trends of the USGS/PNSP database in the USA and is therefore a specific measure of our estimates as a function of active ingredients, crops, and environmental variables used for global scale inference. *QF*_*HT*_ is defined as

7 $$Q{F}_{HT}(i,j)=\left[n(i,j)+\frac{1}{k}\sum _{k}\left|{R}_{{X}_{k}}(i,j)\right|\right]/2$$

where the relative number of existing points *n*(*i*,*j*) and the correlation strength *R*_*x*_(*i*,*j*) of APR of active ingredient *i* on crop *j* against the environmental variable *X* in the USGS/PNSP are defined in Eq. (4). *QF*_*HT*_ is a matrix of scalars between 0 and 1 and complements *QF*_*SA*_.

Finally, *QF*_*Z*_ (Fig. 1, step 14) accounts for country-specific global-scale validation quality against the FAOSTAT pesticide database. Validation of estimates described in “Methods” was conducted on the accumulated mass *M* of country-specific FAOSTAT aggregated data of “herbicides”, “insecticides”, and “bactericides and fungicides”. The related quality factors for country *c* existing in the FAOSTAT are defined as

8 $$Q{F}_{Z}(c)=1-\frac{\left|{M}_{Z}(c)\,-\,{M}_{Z,FAO}(c)\right|}{{M}_{Z}(c)+{M}_{Z,FAO}(c)}$$

where *Z* is one of “herbicides”, “insecticides” or “bactericides and fungicides”. These quality factors are scalars between 0 and 1, meaning that the higher the *QF*_*Z*_ value, the higher the quality of validation (i.e., smaller estimate error).

All quality factors were tracked all the way down to the final estimates in Fig. 1, step 14, and were ultimately factorized in the quality index *QI* defined by the geometric average

9 $$QI(i,j,c)={(Q{F}_{SA}(j)\cdot Q{F}_{HT}(i,j)\cdot \prod _{Z}Q{F}_{Z}(c))}^{1/(2+z)}$$

The quality index for active ingredients that belonged to multiple pesticide classes was explicitly accounted for in Eq. (9) in the extension *z* of the product term, that is, if active ingredient *i* is both an herbicide and insecticide, then *QI* is the geometric average of four quality factors with the latter two being described in the product term with z = 2. For active ingredients not classified into any of those categories, *QI* in Eq. (9) only includes *QF*_*SA*_ and *QF*_*HT*_ with the power 1/(2 + *z*) = 1/2 with z = 0. The quality index *QI* is globally gridded at the same 5 arc-min resolution of estimated maps of application rates and is distributed in PEST-CHEMGRIDS together with the application rates specific for each crop and active ingredient in the same file formats. An example of a quality index map is provided in Fig. 4 relative to glyphosate on corn.

### Limitations of methods of estimation

We identify a number of limitations that affected PEST-CHEMGRIDS estimates, which include sample size and the use of first-order polynomials for time projections and spatial inference. The sample size introduces uncertainty in the quality of time projections and spatial inferences. We have not conducted a comprehensive global sensitivity analysis on the method structure but assessment tests described in “Validation of historical trends and projection prefactors” and “Validation of spatial inference methods” were satisfactory to our purposes. Aleatory components such as pesticide trading, geopolitical interactions (diplomacy/embargoes/conflicts), and socioeconomic influences (e.g., regulatory decisions, environmental protection movements, and consumers choices) on the use of specific ingredients or classes of ingredients were not explicitly taken into account and may require some causative factor currently excluded in our methods.

One aspect that is thought to contribute uncertainty in PEST-CHEMGRIDS is that the FAOSTAT database lacks for a number of reporting countries relative to pesticides, and we currently have no additional or better information to use to the purpose of conditioning our estimates. Our assumption of “proximity” implemented in Section “Global conditioning against FAOSTAT pesticide records” follows therefore the principle of geophysical vicinity highlighted in^50^ and^59^.

A lack of accurate databases of bans and biotechnologies involving GM as well as their chronology is the most limiting information that prevents us from producing reliable reconstructions of historical use of pesticides. However, we intend to maintain the PEST-CHEMGRIDS and include reconstructions should new research be undertaken to build missing chronologies.

Designing systematic criteria to quantify goodness of estimates of individual ingredients beyond the validation tests proposed here is generally difficult; first, aggregated crops used in PEST-CHEMGRIDS integrate a number of individual crops types that generally undergo a different number of applications and, second, the different crops can receive different application rates per treatment. In contrast, PEST-CHEMGRIDS provides the integral annual application rates in this first release. Future improvements may include constraints on individual ingredients such as number of applications and rates per treatment in order to improve estimate quality.

We have conditioned our estimates based on near-current (2015) agricultural practices, but we have not included an accounting of special farming practices such as organic, biodynamic and similar. Recent studies have brought evidence that an increasing number of farmers in high-income countries are progressively transitioning to agriculture with limited use of synthetic agrochemicals. It is therefore possible that some active ingredients will see a decline in the coming decade in some regions.

There are a number of other factors that we did not include in the generation of projections such as potential climatic shifts, changes in population habits and attitudes to foods, and changes in diets, which are not expanded here. However, all of our estimates provide an expected range in application rates (denoted by high “HIGH” and low “LOW” rates) and we assumed that aleatory fluctuations in application rates can be contained within those ranges.

## Usage Notes

The PEST-CHEMGRIDS data release is the first of its kind and we plan to continue its maintenance as well as expand on active ingredients, increase resolution and metadata, and validations. For the purpose of data reuse, we distribute a basic customizable script written in Matlab 2018a that helps the user to read and convert data in other preferred formats. However, data can be read in any computational environment that is compatible for .TIFF/.TIF and .NC formats of georeferenced maps. Software for further processing PEST-CHEMGRIDS maps includes licenced Mathworks Matlab and Arcgis and freeware software such as QGIS. We are willing to provide guidance with software that we are familiar with upon reasonable request. For questions, collaborations or suggestions, please contact the corresponding author.

## ISA-Tab metadata file

Download metadata file

## Data Availability

All data elaborated from original sources and newly produced in this work were the result of custom-built codes written in Mathworks Matlab2018a on Windows PC. These consist of several independent and dependent scripts and functions to read and reorganize variables, perform calculations, and save intermediate and final data. Custom scripts were also developed to generate .PNG images, georeferenced .TIF maps, and georeferenced NetCDF4 .NC maps of both application rates and data quality. Due to the complexity and size of source data, which use several storage formats and occupy approximately 43 GB, scripts are not directly distributed but are available along with all source data upon request. The only custom code that is distributed with PEST-CHEMGRIDS is the script written in Matlab2018a to read any of the two data storage formats and redraw figures of application rates and their quality indices.
